# Exclusive breastfeeding: measurement and indicators

**DOI:** 10.1186/1746-4358-9-18

**Published:** 2014-10-20

**Authors:** Ted Greiner

**Affiliations:** 1 Department of Food and Nutrition, Hanyang University, 222 Wangsimni Ro, Seongdong-gu, Seoul 133-791, South Korea

**Keywords:** Exclusive breastfeeding, Indicators, Since birth, Current status, Point-in-time, Life-long, Survey methodology, Infant feeding

## Abstract

**Background:**

Accurate measurement of the duration of exclusive breastfeeding is complicated by factors related to definitions, timing, duration of recall, methods of analysis, and sample biases. Clearly prospective methods are likely to be more accurate but are too expensive to use in most large-scale surveys. Internationally, most surveys use a point-in-time or current status measurement (usually 24-hour recall) and report their findings using an indicator established by the World Health Organisation (WHO) in 1991 that involves combining all babies less than six months old in order to obtain a large enough sample size to result in stable proportions that can be compared over time. However, this indicator is complex to understand and explain and is widely misunderstood, even within the breastfeeding community. It is commonly cited in ways that greatly exaggerate how common exclusive breastfeeding actually is.

**Discussion:**

A life-long or since birth indicator, introduced in 2000, counts infants as no longer exclusively breastfed as soon as anything else is fed to them. This is appropriate to do if for example data are being used to link infant feeding patterns with vertical transmission of HIV or later patterns of infant allergy. However, this indicator underestimates the total extent of exclusive breastfeeding, since some women interrupt but then resume it after a period of supplementation (which could for example only be a small amount of water given a single time).

**Summary:**

Exactly which indicator is best to use depends on the purpose for which the data are being used. However, for surveys, the best approach, rarely used, would be to report indicators based on both point-in-time and life-long data.

## Background

The World Health Organization (WHO) began to recommend that babies be exclusively breastfed in 1990 [1], and since 2001 has stated that the optimal duration is six months (180 days) [2]. However, researchers often find it difficult to obtain data on exclusive breastfeeding, forcing some to report only on rates of “predominant” (only non-milk fluids given in addition to breast milk) and “partial” (breastfeeding combined with other milks and/or solid foods) [3].

Confusion on how to measure and even how to report the duration of exclusive breastfeeding is still common, even in the “breastfeeding community”. In the Action Folder for the 20^th^ World Breastfeeding Week in 2012 [4], one finds the following wording: “less than 40% of babies benefit from exclusive breastfeeding for six months”. Of course this statement would be correct if anything less than 40% did so, but globally it is likely that a very low proportion of women continuously exclusively breastfeed “for” six months. In the abstract of a paper in the WHO Bulletin, we have the following: “. . . only 28.7% of infants younger than 6 months had been exclusively breastfed”. [5] Would one day have been adequate to be included in this percentage?

What the authors above are actually referring to is probably the percent of all babies in the age range 0 - 5.99 months who received no food or fluid but breast milk the day before a questionnaire was administered to their mothers. This is the way WHO originally advised that data on exclusive breastfeeding be reported [6]. Thus a 24-hour recall is done with mothers of all infants 0 - 5.99 months of age to ask what they fed the infant the day before the survey. The number who said “nothing but breast milk” is divided by the total number, resulting in a proportion (usually turned into a percentage).

However, the resulting proportions represent a substantial exaggeration if assumed to represent how many babies are exclusively breastfed for the entire recommended six-month period. Several researchers have commented on this problem, as summarized by Agampodi et al. [7] and as discussed cogently by Hector [8] and by Noel-Weiss et al. [9]. The implications of the use of different breastfeeding definitions in studying infant growth were explored by Piwoz et al. [10]. In 2010 , WHO and others issued part 2 of a document on indicators for assessing infant and young child feeding which advised researchers to report on the proportion exclusively breastfeeding in smaller age groups if sample sizes allowed [11].

The goal of this paper is to explore methods of measuring exclusive breastfeeding and to argue that large-scale surveys should report both life-long data, that is, the proportion exclusively breastfeeding continuously from birth to any given age, and the point-in-time (usually 24-hour recall) proportion at that same age (or age group).

## Discussion

### Definitions

Exclusive breastfeeding, though likely practiced widely in the distant past, was no longer traditional by the time the feasibility of giving it a blanket recommendation was discovered and reported on [12] (until that time, it was widely assumed that, at least in hot climates, breastfed babies needed additional water, so it was not possible to give a blanket recommendation to exclusively breastfeed, according to the modern WHO definition that excludes water). Health workers, let alone mothers, had no conception of what it actually was and definitions were widely different even among researchers and breastfeeding specialists, certainly until a seminal paper on breastfeeding definitions was published [13]. Even now, varying definitions are commonly used, with the most common divergence from the WHO definition [6] being the inclusion of water [14].

Thus simply asking mothers how long they breastfed exclusively will rarely provide valid data. In one example [15], a definition was given only when the mother asked for it, likely resulting in data based on varying definitions. This could explain the unusually high proportion of infants reported to have exclusively breastfed for six months (31% of those raised by their biological mother) in this study [15].

This problem can be eliminated by asking mothers either what they gave yesterday (usually in the previous 24 hours; point-in-time) or, to obtain life-long data, the age at which the infant first received each of a long list of locally fed liquids or solids in addition to breast milk (followed by a question whether anything else was given and at what age it was introduced). The age when the first supplement of any kind was given, including water, provides the duration of exclusive breastfeeding. The age at which milk or solid foods was first added then provides the duration of predominant breastfeeding.

In most cultures, any life-long indicator will underestimate how much effective exclusive breastfeeding is going on, since a baby leaves the exclusive breastfeeding category as soon as anything else is introduced, even if this was a one-time phenomenon. In reality, many babies may shift back and forth from being exclusively to predominantly to partially breastfed [16].

There will presumably not be many research objectives that would justify the effort required to record the cumulative number of days each baby has been fed each of these ways and I have not come across any. Certainly for large scale surveys, this would be too complex; for retrospective research the level of accuracy that could be obtained would be too low to achieve required levels of reliability and validity.

An additional definitional problem is that in many cultures most babies receive so-called “prelacteal feeds” in the first 2-3 days of life even if they then revert, perhaps for several months, to strict exclusive breastfeeding. In such a setting, a life-long indicator will greatly underestimate exclusive breastfeeding for many purposes, indicating that most babies received none at all. Thus, for some purposes perhaps the best indicator might be “continuously exclusively breastfed since seven days of life” which would ignore those who received prelacteal feeds. Adding further complexity, breast milk may be expressed or donor milk provided and thus an exclusively breast-milk fed baby may not be fed entirely directly from the mother’s breast [9,17].

### Timing

#### *Point-in-time data*

The point-in-time method has an obvious advantage in avoiding the risk of recall error, but by definition sampling is limited to mothers with children under six months of age. Since so few babies are exactly six months old at the time of any survey, the 24-hour recall method cannot be used to estimate how many babies are still exclusively breastfed at exactly six months of age. Measuring the proportion of babies who are currently exclusively breastfeeding between five and six months of age would result in only a slight overestimate. Some babies 5.0 to 5.9 months of age may stop exclusive breastfeeding some days or weeks after the survey. Thus the proportion doing so for a full six months will be slightly less than what is estimated by looking at all babies 5.0-5.99 months of age.

A survey of say 3000 children under five years of age (a common age range covered in, for example, the DHS—Demographic and Health--surveys), would offer a sample size of only 50 - 60 infants five to six months of age, too small to provide an estimate stable enough to examine annual trends for example. Some national surveys do overcome this either by having a much larger sample size or by oversampling infants and may thus sometimes arrive at a sample of 5.0-5.99 month infants ten times this large, allowing the use of a one-month age interval in arriving at a stable estimate of exclusive breastfeeding continuing until six months of age. This is perhaps the only way to reliably use the point-in-time indicator to provide a reliable estimate of the proportion of infants exclusively breastfed “at” (still not quite “for”) six months.

More common is still to combine all babies currently under six months of age (sometimes estimates are based on all babies under four months of age which of course will yield an even higher percentage). The average age of the babies in a 0 - 6 months age group is clearly going to be about three months of age. Even in low-income countries where many babies may receive little if anything else for the first 2 - 4 months of life, after that age, the nearly universal “traditional” pattern of infant feeding was to introduce supplements well before 6 months of age. Thus variations in national durations of exclusive breastfeeding reported using this WHO indicator are heavily dependent on the extent of exclusive breastfeeding in the first 2 - 3 months. Nevertheless, the WHO indicator is useful and allows simple comparison among surveys not conducted on very large sample sizes; it is the misrepresentation and misreporting of this easily misunderstood indicator that is a major problem.

For cost reasons, most national surveys are cross-sectional and thus will conduct a 24-hour recall only once. Even prospective research often uses repeat 24 hour recalls instead of asking whether any other food or fluid was introduced since the last questionnaire was administered. Some research uses a 7-day diary to obtain detailed data on the current feeding pattern [18] but clearly this is not feasible for most surveys.

#### *Retrospective data*

While the duration of any breastfeeding tends to be recalled accurately even years later, recall of the ages when various other foods and fluids were introduced is much less accurate [19]. Bland et al. [20] used a detailed method to compare the duration of exclusive breastfeeding as obtained from a 48-hour recall with a recall 7 - 9 months later, finding that the latter tended to exaggerate the duration. This problem is likely becoming worse in areas where exclusive breastfeeding is being widely promoted due to the social desirability bias. In a recent Boston hospital sample followed prospectively for 3.5 years, Burnham et al. [21] found that only 30% reported a duration of exclusive breastfeeding two years later that matched the duration obtained prospectively. Though only 0.7% had exclusively breastfed for six months, 22% reported doing so in the later questionnaire. Only 4% underestimated the duration two years later [21].

Recall periods in large-scale retrospective surveys vary, and may do so even within the sample. For example, in a recent national survey in the USA [22], the average recall period was about 7 months but could be up to 13 months.

Recall errors will certainly be smaller the shorter the recall period is. Thus the optimal approach for obtaining life-long data on exclusive breastfeeding might be to interview all mothers with babies less than 7 (or perhaps 9) months of age and ask them for each of a comprehensive list of locally used early supplements when it was first given to the baby. Since many infants will still be exclusively breastfed when the mother is interviewed, life table or survival approaches must then be used to make full use of all data in such an analysis. In using this method, the current age is used as the duration of exclusive breastfeeding for those who have not yet introduced anything and they are dropped from the denominator at ages beyond their current age at the time of their interview. Since these babies will continue to be exclusively breastfed for some unknown period of time after their interviews, this approach somewhat underestimates the duration of life-long exclusive breastfeeding. Several studies have been based on obtaining retrospective data from representative samples of infants in a given geographical area less than seven months of age and using life-table methods to analyze the data [23-25].

### Other methodological problems

In the UK, a national survey of feeding practices is conducted every five years via postal questionnaires. The duration of exclusive breastfeeding is derived from responses to questions about the age at which various foods were introduced from repeated responses at birth, 6 weeks, and 2, 3, 4, and 6 months [26]. Thus recall periods are quite short, but the accuracy of the data is still uncertain due to low response rates from younger mothers and those with lower socioeconomic status. The duration of exclusive breastfeeding has varied from 65% at birth to <1% “at” 6 months [26]. Since exclusive breastfeeding during the seventh month of life (“at” six months of age) is not recommended, perhaps “at 180 days” or “to 6 months” would be a better wording.

Since 2001, the US Centers for Disease Control (CDC), in its breastfeeding questions on its annual national immunization surveys conducted by telephone among mothers with children19 - 35 months old, has asked about the age when any food or fluid was first added to the baby’s diet [27]. Recall bias of the kind reported by Burnham et al. [21] probably largely explains why the CDC reports surprisingly long periods of life-long exclusive breastfeeding, varying from 52% at 7 days to 15% at 6 months (180 days) [28]. But in addition, bias may be increasingly introduced due to the gradual decline in rates of subscription to landline telephones, the basis for sampling, especially in younger and lower income mothers who may now often possess only cell phones and may exclusively breastfeed less than others. However, the CDC has recently checked this and not found much change when a cell phone sample was added.

### Comparison of point-in-time and lifelong data

How much the WHO indicator gives an exaggerated sense of the duration of uninterrupted exclusive breastfeeding will vary from one infant feeding culture to another and may vary over time, possibly making comparisons for the purpose of establishing trends over long periods of time somewhat inaccurate. A first estimate of the extent of this bias came from a large, detailed, prospective study conducted from 1989 - 1994 in Sweden [29]. At that time, Swedish breastfeeding rates had already greatly increased from their lows of the early 70s, and knowledge of and encouragement to exclusively breastfeed were rapidly growing. Women tended to introduce solid foods quite gradually at 4 - 6 months of age, although some gave a relatively large volume of infant formula occasionally before that.

Over 500 women recorded every day the types and amounts of everything they gave their babies for approximately the first nine months of life. This allowed a comparison of how many were currently exclusively breastfeeding at various ages with how many had been doing so the entire time since birth. A total of 92% were exclusively breastfed on their 59^th^ day of life, but only 51% had done so the entire time since birth. At four months, those statistics were 73% and 30% and at 6 months, 11% and 1.8% respectively. Thus the proportional difference between the two approaches increased as infants got older [29]. If one can generalize from this, the extent to which the point-in-time estimate will vary from the life-long estimate increases, the older the infants are.

In Uganda, Engebretsen et al. [30], using a somewhat less precise approach, found that the point-in-time approach estimated the mean duration of exclusive breastfeeding to be about three times as high as life-long data did at both 6 and 12 weeks, though the period of exclusive breastfeeding was quite brief using both approaches.

### Which approach is “best”?

No single indicator in common use will provide a complete and accurate measure of how many days of exclusive breastfeeding infants have received by the age of 180 days. This would be too complex and expensive to obtain and indeed provides unnecessarily precise data for most purposes. For example, we do not weigh humans to a precision of 0.001 grams, although in theory this could probably be done.

In the final analysis, which indicator is “best” depends on the objective of the research or the use to which survey data will be put. Where “ever fed any kind of supplement” would be important, the life-long approach would be best because it permanently knocks a baby out of the exclusive breastfeeding category the first time anything but breast milk (or a prescribed medication) is given to the child. WHO later changed its definition and the use of oral rehydration solution (ORS) is “allowed” in the definition of exclusive breastfeeding. For certain purposes including ORS may not be wise, especially where the water used is likely to be contaminated. Examples where life-long data would be critical to use include linking infant feeding to transmission rates among HIV-exposed infants [31] or examining the relationship between exposure to non-breast milk allergens and the later debut of allergic diseases. The life-long approach might however under-estimate the exclusive breastfeeding of interest if for example one were estimating the link between breastfeeding patterns and infant diarrhea because the latter is likely to respond mainly to fluids and foods given in the past several days.

### Point-in-time versus life-long data in impact evaluation

Exclusive breastfeeding is now widely recognized, measured and promoted. Yet, using the original 0 - 6 month point-in-time WHO indicator, the increase that has taken place after two decades of effort in the number of days babies have been exclusively breastfed probably appears smaller than it actually is. At a meeting in 1992, WHO justified use of the point-in-time 0 - 6 month indicator because it offered a relatively high baseline to start with. At that time, there probably were virtually no infants exclusively breastfed continuously from birth to six months of age and this was thought to risk discouraging policy makers. However, over two decades later, that same high baseline results in it not appearing that much progress has been made. In the developing countries, exclusive breastfeeding has increased on average by nine percentage points, from 34% - 43% over a 22-year period, a rate of improvement of well under 1% per year [32]. If a life-long indicator had been used, rates of exclusive breastfeeding from birth to six months or even to four months would have probably gone up by several times their tiny percentages at baseline, indicating that perhaps a 20% improvement per year had been taking place.

### What to do for surveys?

Each of the two major methods of measuring exclusive breastfeeding skews our perceptions of how much exclusive breastfeeding is going on, but they do so in opposite directions. The WHO indicator made exclusive breastfeeding at “baseline” (the early 90s) appear to be more common than it was. In turn, it is not a very sensitive method to measure the improvement that has occurred since then. For many purposes, the life-long indicator penalizes too much for a single diversion from exclusive breastfeeding (thus it would register that none took place in a society with universal prelacteal feeding, even if most went on to receive 179 days of exclusive breastfeeding). Thus the wisest approach for surveys, the purpose of which must be to provide the most accurate characterization of the duration of exclusive breastfeeding in a given sample, would be to report both point-in-time and life-long data. One simple additional question would allow the life-long indicator to be created. All women who say they gave nothing but breast milk the day before the survey are then asked if they have EVER given anything else. This method has sometimes been used by researchers [33,34]. More accurate would of course be to ask those currently exclusively breastfeeding when any food or fluid was given to the infant in the past. Exclusive breastfeeding ends when anything has been added; the duration of predominant breastfeeding could also be estimated as the first age when any solid food or milk was given. This has also been done by some researchers [35,36].

To obtain life-long data in national surveys would require extra effort and costs. Oversampling among infants younger than perhaps 7 or 9 months might be required; and the life-long data would need to be analyzed using life table or survival techniques [37,38].

## Summary

Accurate measurement of the duration of exclusive breastfeeding is complicated by factors related to definitions, timing, duration of recall, methods of analysis, and sample biases. Clearly prospective methods are likely to be more accurate but are too expensive to use in most large-scale surveys. Internationally, most surveys use a point-in-time or “current status” measurement (usually 24-hour recall) and report their findings using an indicator established by WHO in 1991 that involves combining all babies less than six months old in order to obtain a large enough sample size to result in stable proportions that can be compared over time. However, this indicator is complex to understand and explain and is widely misunderstood, even within the “breastfeeding community”. It is commonly cited in ways that greatly exaggerate how prevalent exclusive breastfeeding actually is—at least that which has occurred continuously since birth. A life-long or “since birth” indicator, introduced in 2000, counts infants as no longer exclusively breastfed as soon as anything else is fed to them. This is appropriate to do if for example data are being used to link infant feeding patterns with vertical transmission of HIV or later patterns of infant allergy. However, this indicator underestimates the total extent of exclusive breastfeeding, since some women interrupt but then resume it after a period of supplementation (which could for example only be a small amount of water given a single time). Exactly which indicator is best to use depends on the purpose for which the data are being used. However, for surveys, the best approach, rarely used, would be to report indicators based on both point-in-time and life-long data.

## Abbreviations

CDC: US Centers for Disease Control; HIV: Human immunodeficiency virus; IBFAN: International baby food action network; ORS: Oral rehydration solution; UK: United Kingdom; USA: United States of America; WABA: World Alliance for Breastfeeding Action; WHO: World Health Organization.

## Competing interests

The author declares that he has no competing interests.

## Author’s information

Ted Greiner has worked on breastfeeding issues at research, program and policy levels, publishing dozens of articles and monographs since 1975, when he conducted the first research to show that advertising influences how infants are fed. In 1978-81 he ran a Rockefeller Foundation breastfeeding program in North Yemen that was gradually followed by a doubling of the duration of breastfeeding. Beginning in 1980, he began arguing for a shift in the existing breastfeeding promotion paradigm, calling for promotion to be subordinated to protection and support. His inputs to and work with the Swedish International Development Cooperation Agency from 1979 to 2004 helped ensure that it has been the only donor maintaining support to IBFAN and WABA (starting in 1992) for the past 35 years. He was integrally involved in the process that led to the Innocenti Declaration in 1990. His presentation to WHO in 1992 and publication of arguments in 1996 effectively ended their use of the ambiguous word “wean”. He was involved in the research that “discovered” exclusive breastfeeding in 1976, in early WHO efforts to define relevant indicators, and was a co-author of the first article in 2000 that showed the importance of the “since birth” or life-long indicator for exclusive breastfeeding.
